# Protein tyrosine phosphatase receptor type O (Ptpro) regulates cerebellar formation during zebrafish development through modulating Fgf signaling

**DOI:** 10.1007/s00018-013-1259-7

**Published:** 2013-01-30

**Authors:** Wei-Hao Liao, Chia-Hsiung Cheng, Kuo-Sheng Hung, Wen-Ta Chiu, Gen-Der Chen, Pung-Pung Hwang, Sheng-Ping L. Hwang, Yung-Shu Kuan, Chang-Jen Huang

**Affiliations:** 1 Graduate Institute of Life Sciences, National Defense Medical Center, Taipei, 104 Taiwan; 2 Institute of Biological Chemistry, Academia Sinica, 128 Academia Rd, Sec 2, Taipei, 115 Taiwan; 3 Department of Neurosurgery, Taipei Medical University-Wan Fang Hospital, Taipei, 116 Taiwan; 4 Institute of Cellular and Organismic Biology, Academia Sinica, Taipei, 115 Taiwan; 5 Institute of Biochemical Sciences, National Taiwan University, Taipei, 106 Taiwan; 6 Neuroscience Program, Academia Sinica, Taipei, 115 Taiwan

**Keywords:** *ptpro*, Cerebellum, *fgf*, *fgfr*, Zebrafish

## Abstract

Protein activities controlled by receptor protein tyrosine phosphatases (RPTPs) play comparably important roles in transducing cell surface signals into the cytoplasm by protein tyrosine kinases. Previous studies showed that several RPTPs are involved in neuronal generation, migration, and axon guidance in *Drosophila*, and the vertebrate hippocampus, retina, and developing limbs. However, whether the protein tyrosine phosphatase type O (*ptpro*), one kind of RPTP, participates in regulating vertebrate brain development is largely unknown. We isolated the zebrafish *ptpro* gene and found that its transcripts are primarily expressed in the embryonic and adult central nervous system. Depletion of zebrafish embryonic Ptpro by antisense morpholino oligonucleotide knockdown resulted in prominent defects in the forebrain and cerebellum, and the injected larvae died on the 4th day post-fertilization (dpf). We further investigated the function of *ptpro* in cerebellar development and found that the expression of *ephrin*-*A5b* (*efnA5b*), a Fgf signaling induced cerebellum patterning factor, was decreased while the expression of *dusp6*, a negative-feedback gene of Fgf signaling in the midbrain-hindbrain boundary region, was notably induced in *ptpro* morphants. Further analyses demonstrated that cerebellar defects of *ptpro* morphants were partially rescued by inhibiting Fgf signaling. Moreover, Ptpro physically interacted with the Fgf receptor 1a (Fgfr1a) and dephosphorylated Fgfr1a in a dose-dependant manner. Therefore, our findings demonstrate that Ptpro activity is required for patterning the zebrafish embryonic brain. Specifically, Ptpro regulates cerebellar formation during zebrafish development through modulating Fgf signaling.

**Electronic supplementary material:**

The online version of this article (doi:10.1007/s00018-013-1259-7) contains supplementary material, which is available to authorized users.

## Introduction

Tyrosine residue phosphorylation by protein-tyrosine kinases (PTKs) is one of the key post-translational modification strategies to switch protein activities on or off by all eukaryotic cells. After the first tyrosine kinase was discovered to regulate protein activities in the 1980s, it was 8 years later before researchers began to uncover that protein-tyrosine phosphatases (PTPs) can regulate protein activities in an opposite but equally important manner as do PTKs [1, 2].

Classical PTPs are defined by the CX_5_R signature motif, and are subdivided into receptor-type PTPs (RPTPs) and non-RPTPs based on their structure and cellular localization. The RPTPs are comprised of cytoplasmic, transmembrane, and extracellular domains. These phosphatases are further classified into eight subgroups (R1–R8 subtypes) according to the specific domain of their extracellular region [1, 2]. Among these phosphatases, the R3 subtype of RPTPs, including PTPRO, DEP1 (CD148), SAP-1, and VE-PTP, shares a similar structure that is characterized by a single catalytic domain in the cytoplasmic domain and several fibronectin type III-like domains in the extracellular region [3]. Prior studies showed that tyrosine phosphorylation catalyzed by Src family kinases (SFKs) occurs in the carboxy-terminal region of several R3 family members, and such phosphorylation of tyrosine residues in these R3 subtype RPTPs promotes the binding of SFKs to RPTPs. Subsequently, dephosphorylation of SFKs by R3 family RPTPs controls activation of SFKs during cell morphological changes [4, 5]. In addition, a study by Shintani et al. [6] demonstrated that dephosphorylation of ephA and ephB receptors by PTPRO, one of the R3 subtype RPTPs, negatively regulates signal transmission through both receptors. Furthermore, using yeast two-hybrid screening and biochemical assays, Chen and Bixby [7] identified and confirmed that PTPRO dephosphorylates one of the NPCD (pentraxin with a chromo domain) isoforms in vitro. An interesting discovery by Kim et al. [8] reported that canonical Wnt signaling induces the expression of *ptpro* transcripts, and an in vitro assay showed that Wnt molecules interact with the *ptpro* extracellular domain. Nevertheless, the molecular functions of these RPTPs, such as the in vivo ligands and substrates for these R3 subtype RPTPs, are still largely unknown.

Functions of R3 family RPTPs during both invertebrate and vertebrate development began to be elucidated by several research groups in the past two decades. The R3 subtype member, PTPRO, was originally identified as a membrane protein called GLEPP1 (glomerular epithelial protein 1) that is expressed by podocytes and brain tissues in rabbit [9]. A subsequent study of *ptpro* knockout mice indicated that PTPRO plays a role in regulating the glomerular pressure/filtration rate relationship in the kidneys through effects on the structure and function of podocytes [10]. In *Drosophila*, the PTPRO homolog, PTP10D, and other R3 subtype RPTPs were shown to regulate neuronal axon outgrowth and guidance [11–13]. The involvement of PTPRO in vertebrate neural development was later elucidated by collective efforts from several reports. For example, Chen and Bixby found that dephosphorylation of NPCD by PTPRO was required for nerve growth factor-induced process outgrowth in mice, and analyses of *ptpro* knockout mice revealed neurogenesis and neuronal pathfinding defects during dorsal root ganglion (DRG) development [7]. In addition, two research groups demonstrated that PTPRO regulated axon outgrowth and guidance in the embryonic chick lumbar spinal cord and retinotectal projection system [6, 14–16]. However, our knowledge of the roles and the operational mechanisms that PTPRO plays during the development of other parts of the vertebrate brain and the contributions of each different *ptpro* isoforms in these developmental events are far from established.

The *ptpro* gene of zebrafish (Ptpro for zebrafish protein) was identified previously, and its expression patterns were characterized during several early embryonic developmental stages [17]. In this report, we concentrated on examining functions of the full-length *ptpro* isoform in zebrafish embryonic development, and provide additional expression analyses of *ptpro* transcripts in different embryonic stages and adult tissues. We conducted a loss-of-function study using an antisense morpholino oligonucleotide (MO) knockdown strategy. The results indicated that injected morphants lacking Ptpro activity exhibited prominent defects in the embryonic forebrain and cerebellum.

The relationship between the function of Fgf signaling and the cerebellum development has previously been shown before. Studies of mice and zebrafish demonstrated that Fgf signaling mediated the function of isthmic organizer (IsO) by activating the *ephrin A* expression to coordinate the development of embryonic cerebellum [18–22]. In addition, the receptors for Fgf ligands (Fgfrs) were shown to activate the transduction of Fgf signaling by self-phosphorylating their own cytoplasmic domains [23, 24]. The shared cerebellum defects in *fgf8* and *ptpro* morphants, and the requisite phosphorylation of Fgfrs for activating Fgf signaling prompted us to study the relationship between Ptpro and Fgf signaling during cerebellum development. Further analyses revealed that the expression of the *ephrin*-*A5b* (*efnA5b*) was decreased while the expression of Fgf signaling induced negative-feedback gene, the *dusp6*, was notably increased in the midbrain-hindbrain boundary (MHB) region in *ptpro* morphants. Subsequent analyses demonstrated that the cerebellar phenotype in *ptpro* morphants could be partially rescued by perturbing Fgf signaling activity with Fgfr inhibitors.

To understand the possible regulatory mechanism of *ptpro* during cerebellar development, we performed affinity pull-down assays and evaluated the tyrosyl phosphorylation level of Fgfr1a. Results showed that Ptpro physically interacts with Fgfr1a and dephosphorylates Fgfr1a in vitro in a dose-dependant manner. Therefore, our experimental results demonstrated that Ptpro activity is required for controlling zebrafish embryonic brain development. Specifically, our analyses suggested that modulation of Fgfr turnover in plasma membranes by Ptpro is crucial for the development of the cerebellum in the embryonic brain.

## Results

### Zebrafish *ptpro* is expressed in embryonic and adult brains

Previous reports indicated that the zebrafish genome encodes one *ptpro* gene (NM_001083814.1) [17]. The existence of mammalian *ptpro* splicing isoforms has been reported previously but whether any *ptpro* splicing isoform exists in zebrafish still remains to be clarified [3, 25]. In this report, we focused on the expression and biological function of the full-length *ptpro* during zebrafish development. The alignment of the zebrafish Ptpro protein sequence between mouse and human PTPRO sequences is showed in Supplemental Figure 1. Alignment comparison showed that the zebrafish Ptpro exhibited 56 % identity (73 % similarity) with mouse and 55 % identity (72 % similarity) with human PTPROs, respectively.

**Fig. 1:**
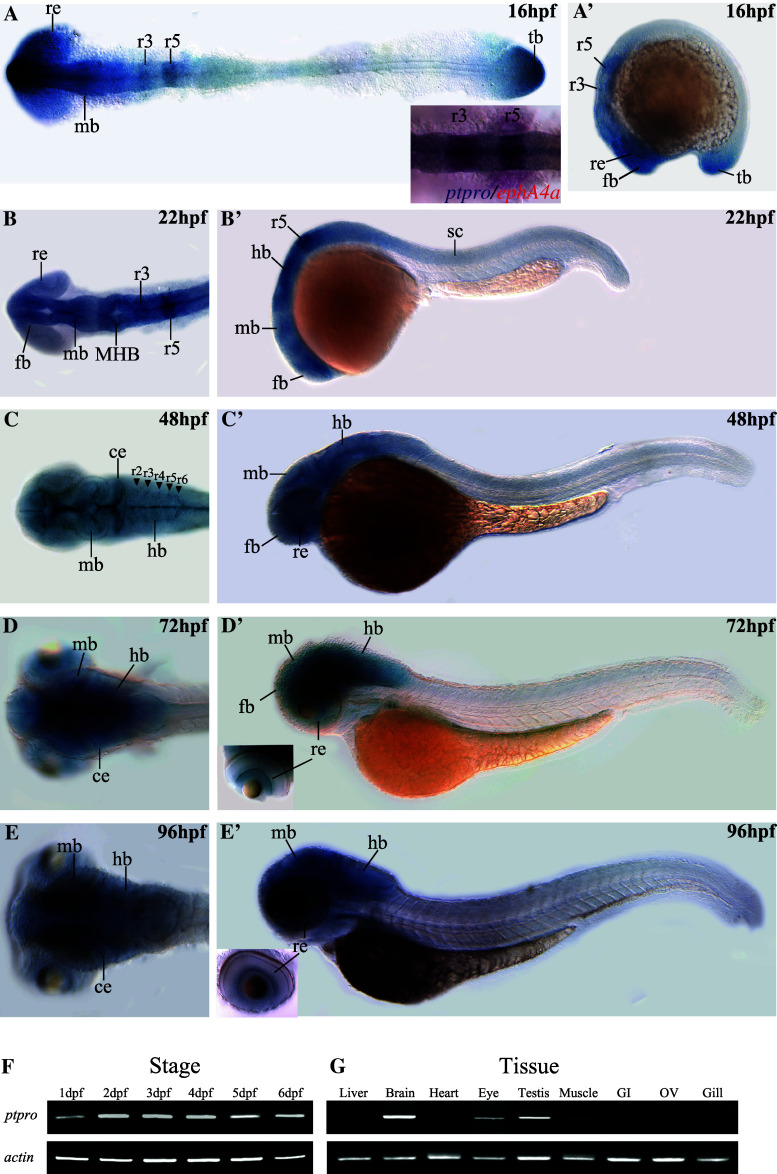
Spatial and temporal expression patterns of the zebrafish *ptpro* gene. Expressions of *ptpro* mRNAs were detected by antisense RNA whole-mount in situ hybridization (*WISH*). Images showing dorsal (**a**–**d**) or lateral views (**a**′–**d**′) of embryos collected at 16, 22, 48, 72, and 96 h post-fertilization (hpf). Anterior side is to the left and dorsal side is to the top. Small inserted image in (**a**) is from our double in situ staining to demonstrate the co-localization of the *ptpro* and *ephA4a* transcripts in the rhombomere 3 and 5. (**f**, **g**) Images of RT-PCR results for *ptpro* transcripts obtained from embryos at different developmental stages (**f**) or different adult tissues (**g**). Lower panels show RT-PCR results of α-actin for the controls. *ce* cerebellum, *fb* forebrain, *hb* hindbrain, *MHB* midbrain-hindbrain boundary, *mb* midbrain, *re* retina, *r3/5* rhombomere 3/5, *tb* tailbud

Expression patterns of *ptpro* in zebrafish embryos at various early stages such as 8-cell, 6 hpf (hours post-fertilization), 10 hpf, 24 hpf, 48 hpf and 72 hpf were previously reported, but its expression in embryos after 72 hpf and in adult tissues has not been described [17]. Because the riboprobe which recognizes the phosphatase catalytic domain may simultaneously detect different *ptpro* isoforms [17], we analyzed the expression pattern of *ptpro* again using riboprobes specific for the extracellular domain sequence of *ptpro* mRNAs. Therefore, the patterns documented in this report represent only the patterns of receptor-type *ptpro* transcripts. During the segmentation stage, stronger expression of *ptpro* was observed in the forebrain, midbrain, rhombomere 3/5 (r3/5), retina, and tailbud, while weaker expression was present in the spinal cord at 16 hpf (Fig. 1a, a′). The smaller image inserted in Fig. 1a (bottom right corner) showing our result from double in situ staining to confirm the co-localization of *ptpro* and *ephA4a* transcripts in the r3/5. At 22 hpf, *ptpro* was continuously expressed in the entire brain, particularly stronger in the MHB and r3/5 and weaker in the spinal cord and retina (Fig. 1b, b′). The distinguishable presence of *ptpro* transcripts in the r3/5 in our analyses was not observed in previously published expression data of *ptpro* at 24 hpf [17]. We reasoned that it might due to the slight differences between the stages of embryos or the probe sequences that each laboratory selected.

From 48 hpf onward to 96 hpf, *ptpro* expression was still predominantly observed in the entire brain (Fig. 1c–e, c′–e′). This is similar to the results published previously [17], except that our result of 48 hpf embryos exhibited notably higher levels of *ptpro* in the midline, cerebellum, and hindbrain segment centers (Fig. 1c, arrowheads). We reasoned that it might be caused by the differences between the focal planes of images, or the probe sequences that each laboratory selected. Beside the brain, *ptpro* expression was detected in the retina at 72–96 hpf (Fig. 1d′, e′). In addition, we performed RT-PCR analyses to investigate expression levels of *ptpro* transcripts during different developmental stages and in adult zebrafish tissues. Our results indicated that *ptpro* was continually expressed at 1–6 dpf (Fig. 1f) and was detected in the brain, eyes, and testes in adult tissues (Fig. 1g). Taken together, *ptpro* was predominately expressed in the brain tissues, implying that it may regulate brain patterning during zebrafish development.

### Knockdown of *ptpro* expression caused prominent developmental defects in the CNS

**Fig. 2:**
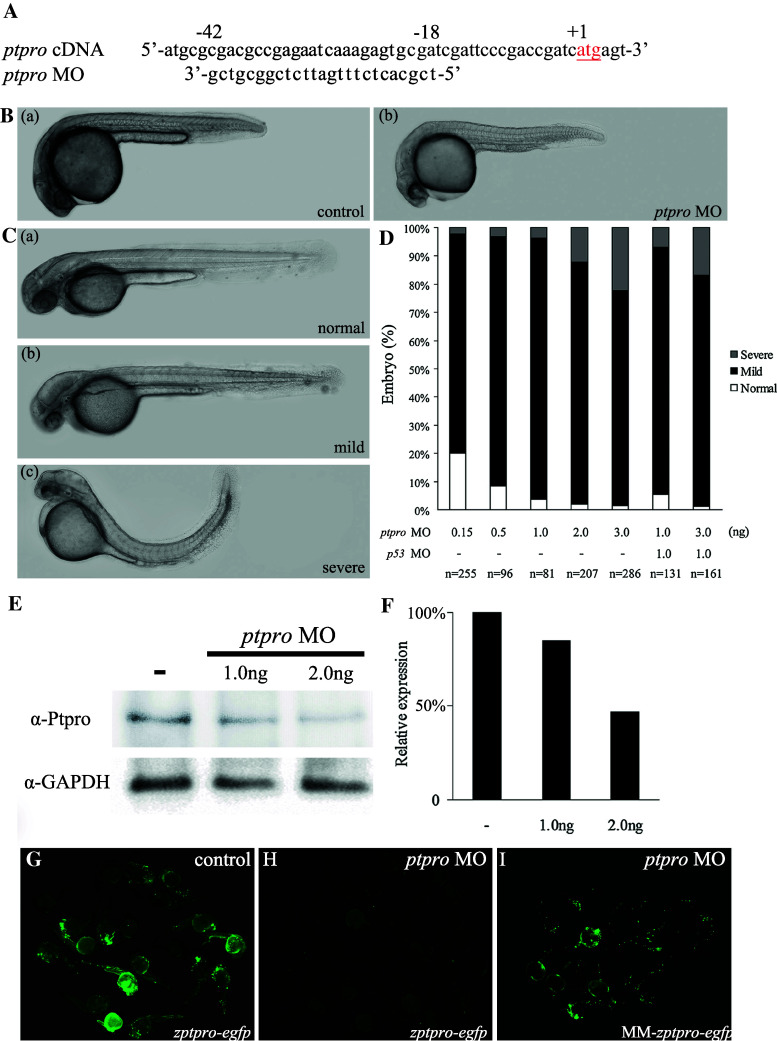
Inhibition of *ptpro* translation caused developmental defects in the central nervous system. **A** Sequence around the translation start site (*in red*) of zebrafish *ptpro* cDNA and the corresponding sequence of *ptpro* MO. **B** Representative images showing control (*panel a*) or MO-injected embryos (*panel b*) at 24 hpf. The head is always to the *left*. **C** Representative images showing normal (*panel a*), mild (*panel b*), and severe (*panel c*) phenotypes at 48 hpf. **D** Chart showing the statistical analysis of embryos injected with various doses of the *ptpro* MO or *p53* MO. **E** Western blot of embryos injected with different doses of MOs against *ptpro* at 24 hpf using an anti-ptpro antibody. GAPDH was used as the loading control. **F** Quantitative analysis of ptpro protein levels in *ptpro* morphants. Values from treated *ptpro* MOs were normalized to matched GAPDH measurements and then expressed as a ratio of normalized values to the control. **G**–**I** Images showing results from MO effectiveness tests using the corresponding *zptpro*-*egfp* expression construct with a control (G), with the *ptpro* MO (**H**), or using the mismatched construct, MM-*zptpro*-*egfp*, with a *ptpro* MO (**I**). Construct descriptions are given in “Materials and methods”. Images were taken at 24 hpf. All embryos were injected with either 1 nl 1 % phenol red as injection control or 2 ng MO unless specified otherwise (such as in **D**)

To determine the role of *ptpro* in zebrafish embryonic development, we employed two *ptpro* MOs to inhibit either the translation (MO = MO^atg^, Fig. 2a) or the splicing (MO^SB^, Supplemental Fig. 2) of *ptpro,* and examined the alterations in control and MO-injected embryos. Because embryos injected with either MOs exhibited similar brain phenotypes (Fig. 2B–D and Supplemental Fig. 2B–C), and the MO consistently showed higher efficiency (>90 % abnormal rate at 3 ng) than the MO^SB^ (around 55 % abnormal rate at 3 ng) to induce brain phenotypes under our experimental conditions, we thus adopted the MO throughout our study.

As can be seen in Fig. 2B, the control phenol red injected embryos exhibited no visible alterations during development (Fig. 2B, panel a). In contrast, *ptpro* MO-injected embryos showed the first phenotypic effects of the MO such as brain abnormality at 24 hpf (Fig. 2B, panel b, C). At 24 hpf, *ptpro* MO-injected embryos exhibited a significantly underdeveloped forebrain (Fig. 2B, panel b). At 48 hpf, *ptpro* MO-injected embryos showed hydrocephalus (with an expanded 4th ventricle), pericardial edema, and reduced sizes of the eyes, tectum, and cerebellum (Fig. 2C, panels b and c). At this stage, we distinguished three phenotypic classes (Fig. 2C). Class I embryos had a normal phenotype (Fig. 2C, panel a). Class II embryos predominantly exhibited the hydrocephalus phenotype as described above (Fig. 2B, mild). Class III embryos had markedly curved tails or severe defects in the trunk (Fig. 2B, severe). The percentage of abnormal phenotypes in zebrafish embryos was correlated to the various doses of *ptpro* MO (Fig. 2D). We noticed that the cardiac edema phenotype observed in the MO-injected morphants was not obviously seen in the splicing blocker MO-injected morphants (Refer to the Supplemental Fig. 2). Therefore, the cardiac edema phenotype appears to be a common non-specific defect of MO injections. The *p53* MO was used to evaluate the non-specific apoptotic effect of injecting *ptpro* MO [26]. The percentage of abnormalities in *ptpro* morphants co-injected with the *p53* MO was approximately equal to that with the various doses of morphants. Taken together, our results indicated that injection of the *ptpro* MO resulted in dose-dependent brain phenotypes specifically, and there is no p53-induced apoptotic effect associated with our *ptpro* MO injection.

To gauge the effectiveness of the MO, total proteins were extracted from *ptpro* MO-injected embryos (morphants) and analyzed by Western blotting to test for possible residual Ptpro protein. We found that the expression of the Ptpro was effectively reduced in a dose-dependent manner by the *ptpro* MO (Fig. 2E, F). On the other hand, we generated a *zptpro*-*egfp* reporter plasmid containing the MO target sequence upstream to the GFP cassette to determine the specificities of the *ptpro* MO. *zptpro*-*egfp* plasmid DNA (2 ng) which was injected alone (Fig. 2G) or with 2 ng (Fig. 2H) of the MO at the one- to two-cell stage, and a group of embryos was photographed at 24 hpf. As a control, the target sequence bearing five mismatches was also employed in the MM-*zptpro*-*egfp* construct (Fig. 2I). Results indicated that the *ptpro* MO was effective. Therefore, this result further supports the *ptpro* MO that we adopted having specificity to knock down the expression of Ptpro.

### *ptpro* morphants exhibit defects in neuronal cell fate determination

**Fig. 3:**
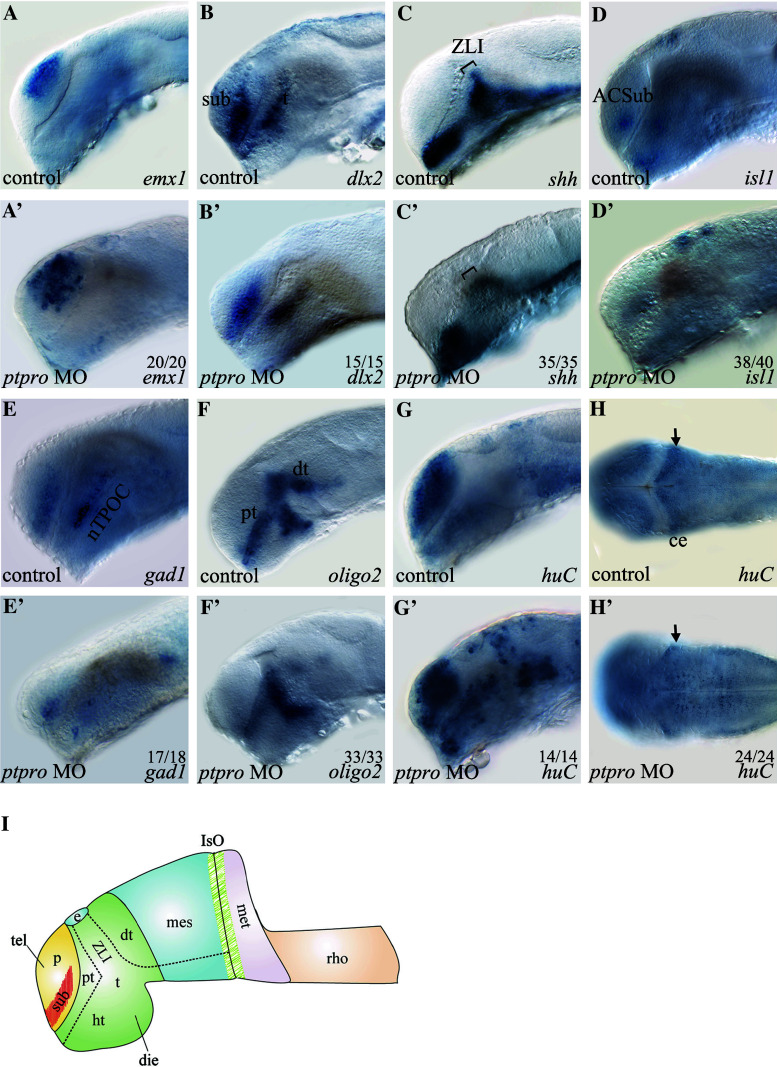
*ptpro* morphants exhibit defects in neuronal cell fate determination. (A-H’) Images of WMISH results from control (**a**–**h**) and *ptpro* MO-injected (**a**′–**h**′) embryos at 24 (**a**–**g**) and (**a**′–**g**′) and 72 hpf (**h**) and (**h**′). Each specific mRNA detected by WMISH is shown in the bottom right corner of each image. Lateral views with the anterior to the left and dorsal to the *top* in (**a**–**g**) and (**a**′–**g**′); dorsal views with the anterior to the *left* and *right* to the *top* in (**h**) and (**h**′). *Arrow* in (**h**′) indicates developing cerebellum. **I** Schematic drawing indicating the locations of dorsal thalamus (*dt*), diencephalon (*die*), epiphysis (*e*), hypothalamus (*ht*), isthmic organizer (*IsO*), metencephalon (*met*), pallial domain (*p*), prethalamus (*pt*), rhombencephalon (*rho*), subpallial domain (*sub*), telencephalon (*tel*), thalamus (*t*), zona limitans intrathalamica (*ZLI*). All embryos were injected with either 1 nl 1 % phenol red as injection control or 2 ng MO. The fraction of embryos displaying each phenotype is labeled on the corresponding image

In order to understand the molecular mechanism that caused the abnormal brain formation in *ptpro* morphants, we analyzed the expressions of several forebrain-midbrain specific differentiation marker genes (*emx1*, *dlx2*, *shh*) and neuronal specification marker genes (*isl1*, *gad1*, *oligo2*, *huC*) in *ptpro* morphants. For reference, we adopted and modified a figure from Wilson and Houart [27] to indicate the locations of these forebrain and midbrain regions (Fig. 3i). Our results showed that while the expression of *emx1* in control-injected embryos is detected in the dorsal pallial of telencephalon, its expression was expanded to the subpallial domain of telencephalon in *ptpro* MO-injected embryos at 24 hpf (Fig. 3a, a′). We next analyzed the expression of *dlx2* that normally is detected in the subpallial domain of the telencephalon and the thalamus. Results showed that injection of the *ptpro* MO reduced *dlx2* expression in the anterior ventral thalamus (prethalamus) and subpallial telencephalon at 24 hpf (Fig. 3b, b′). In addition, the *dlx2* expression pattern in the dorsal thalamus had changed in *ptpro* morphants, implying a developmental defect of the zona limitans intrathalamica (ZLI) in the forebrain, a narrow transverse boundary between the anterior forebrain (telencephalon, hypothalamus, and prethalamus) and diencephalon (thalamus and pretectum) [27, 28]. Previous studies indicated that *shh* is expressed in the embryonic ZLI and was identified to regulate the patterning of prethalamus and dorsal thalamus in the diencephalon [28]. Therefore, we employed the *shh* probe to examine the effects of knocking down PTPRO on the formation of the ZLI. Our results confirmed that at 24 hpf, reduced expression of *shh* within the ZLI region was observed in *ptpro* morphants (Fig. 3c, c′). Taken together, these results demonstrated that inhibiting *ptpro* expression with the MO caused cell-type specification defects in the telencephalon and the telencephalon-diencephalon boundary ZLI.

Besidesspecific brain patterning marker genes, we also examined the expressions of several neuronal differentiation marker genes to evaluate the role of *ptpro* on the development of different neural cell lineages. It was previously shown that the expression of *islet*-*1* (*isl1*) was found in all primary motor neurons (PMNs) in zebrafish [29], and *isl1*-null mice embryos failed to develop motor neurons [30]. Our analyses showed that *ptpro* MO-injected embryos lost *isl1* expression in the anterior commissure of the subpallial telencephalon (ACSub) and in the hypothalamus of the diencephalon (Fig. 3d, d′) suggesting that specification of motor neurons is suppressed in *ptpro* morphants. In zebrafish, *gad1,* a marker for γ-aminobutyric acid (GABA) neurons, is specifically expressed in GABAergic interneurons of the subpallial telencephalon and the nucleus of the tract of the postopic commissure (nTPOC) [31]. Our analysis indicated that *gad1* expression was severely reduced in both the subpallial telencephalon and nTPOC in *ptpro* morphants (Fig. 3e, e′). These data demonstrated that *ptpro* is necessary for appropriate development of GABAergic interneurons in the telencephalon. To investigate the effects of *ptpro* on oligodendrocyte specification, we examined *olig2* expression in *ptpro* morphants because *olig2* is essential for generating motor neurons and forming the oligodendrocyte progenitor during development [32]. Previous studies indicated that *olig2* is expressed in the prethalamus, ventral thalamus, and dorsal thalamus at 24 hpf in zebrafish [32, 33]. Our results show that injecting *ptpro* MO did not affect *olig2* expression in the ventral thalamus, whereas *olig2* expressions in the dorsal thalamus and prethalamus were significantly reduced (Fig. 3f, f′). These results demonstrated that specification of oligodendrocytes in the dorsal thalamus was suppressed in *ptpro* MO-injected embryos. Taken together, knockdown of *ptpro* expression changed forebrain patterning that caused reductions in specifying the motor neurons, GABAergic interneurons, and oligodendrocytes.

Territorial specification is related to cell-type specification within the developing brain. For example, the numbers of specific neurons derived from the subpallial telencephalon and their migration to the pallial telencephalon are linked to neural differentiation during forebrain development [34]. In *ptpro* MO-injected embryos, changes in expressions of forebrain patterning genes implied the influence of neural differentiation during development. We therefore investigated *huC* (*elavl3*) expression, a marker of differentiating neurons including post-mitotic neurons, in *ptpro* morphants. The results showed that the numbers of *huC*-positive cells appeared to markedly increase in the telencephalon and diencephalon (Fig. 3g, g′) but decreased in the developing cerebellum (Fig. 3h, h′) in the *ptpro* morphants. These observations imply a defect in neural differentiation process in *ptpro* morphants because, rather than showing an universal decrease of all markers analyzed, the marker genes such as the *emx1* and *huC* were in fact obviously increased in several brain regions in the *ptpro* morphants (Fig. 3a′, g′) despite that the markers such as the *dlx2*, *shh*, *isl1*, *gad1* and *oligo2* (Fig. 3b, b′, c, c′, d, d′, e, e′, f, f′) were notably decreased in the *ptpro* morphants.

### Ptpro mediates embryonic cerebellar development

**Fig. 4:**
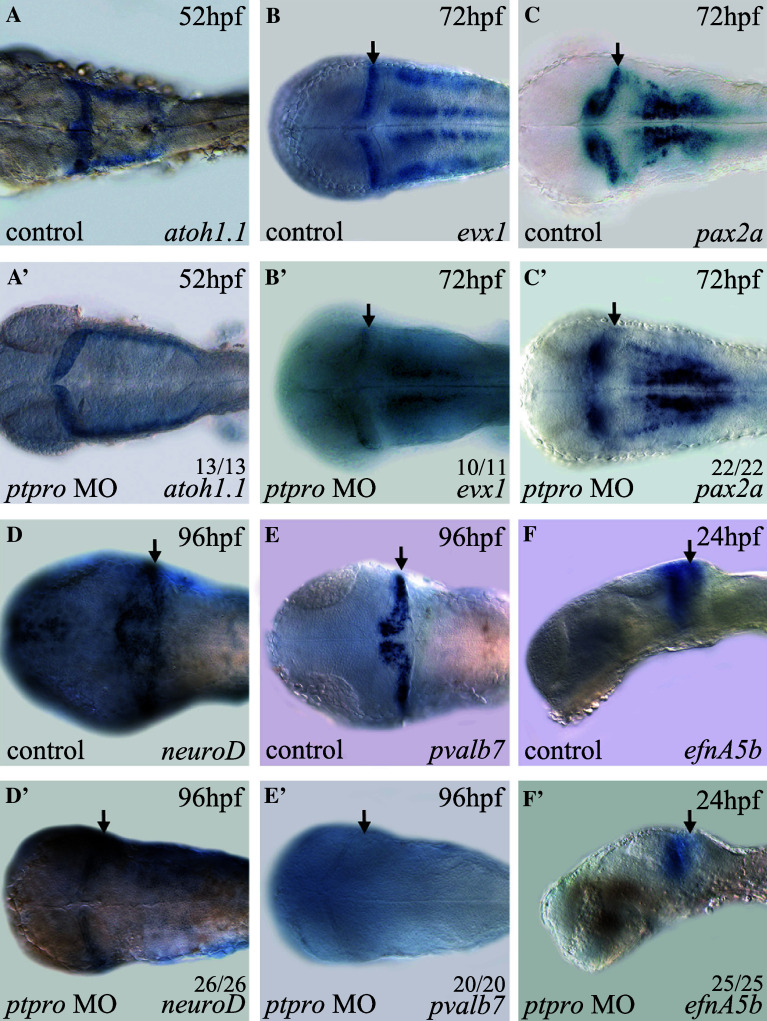
*ptpro* morphants exhibit defects in cerebellar development. (**a**–**f**′) Images of WMISH results from control (**a**-**f**) and *ptpro* MO-injected (**a**′–**f**′) embryos at various stages as indicated in the *top right corner* of each image. Each specific mRNA detected by WMISH is shown in the *bottom right corner* of each image. Dorsal views with the anterior to the *left and right* to the *top* in (**a–e**) and (**a**′–**e**′); lateral views with the anterior to the *left* and dorsal to the *top* in (**f**) and (**f**′). *Arrows* indicate locations of the developing cerebellum. All embryos were injected with either 1 nl 1 % phenol red as injection control or 2 ng MO. The fractions of embryos were labeled on each corresponding image

In our analyses, we noted that there were neuronal specification defects within the MHB and cerebellum of *ptpro* morphants at 24 and 72 hpf (Fig. 3g, g′, h, h′). This cerebellar defect was also reflected by the reduced expression of N-acetylated tubulin (AcTub) in *ptpro* morphants at 72 hpf (data not shown). To further investigate the function of *ptpro* in development of the cerebellum, we decided to examine the expression pattern of *atoh1a* at an early stage, because previous reports showed that *atoh1a*-expressing progenitors in the upper rhombic lip (URL) generated granule cells within the cerebellar compartment in mammals and zebrafish [35–37]. Results from our analysis indicated that despite the 4th ventricle being markedly expanded in *ptpro* morphants, the specification of *atoh1a*-positive cells at 52 hpf was fairly normal in the URL region compared to the injection controls (Fig. 4a, a′). Therefore, our data indicated that loss of Ptpro activity did not affect cerebellar progenitor formation. We subsequently analyzed the expression of the cerebellum patterning genes, *evx1* and *pax2.1*, because they were reported to be expressed in the developing cerebellum [38–40], IsO, and MHB during embryonic brain development [41]. Our results indicated that expressions of *evx1* and *pax2* were specifically reduced within the cerebellar region at 72 hpf in *ptpro* morphants, suggesting the involvement of Ptpro activity in cerebellar development (Fig. 4b, b′, c, c′). To understand the effect of depletion of Ptpro activity on cerebellar development, we evaluated expressions of specific marker genes of the cerebellar granule and Purkinje cells. Previous studies showed that the transcription factor, *neuroD*, is expressed in mouse and zebrafish cerebellar granule cells [21, 35, 42, 43]. In *ptpro* morphants, *neuroD* expression was markedly reduced within the cerebellar compartment at 96 hpf (Fig. 4d′). We also evaluated the effect of the *ptpro* MO on the expression of *Parvalbumin7* (*pvalb7*), a marker of differentiated Purkinje cells in the cerebellum [35, 37]. Results showed that injection of the *ptpro* MO completely abolished *pvalb7* expression in the cerebellum at 96 hpf (Fig. 4e, e′). Taken together, our analyses indicated that granule and Purkinje cells were both reduced within the cerebellum of *ptpro* morphants, suggesting that loss of Ptpro activity in zebrafish embryos specifically suppressed cerebellar formation during development.

To examine the molecular basis of cerebellar defects in *ptpro* morphants, we studied expressions of *ephrin*-*A5a* (*efnA5a*) and *ephrin*-*A5b* (*efnA5b*), because previous reports showed that establishing anterior to posterior gradients of these two *ephrins* controlled by Fgf signaling is required for development of the embryonic cerebellum [20]. In *ptpro* morphants, we found that expressions of *efnA5b* and *efnA5a* were markedly reduced in the anterior metencephalon at 24 hpf (Fig. 4f′, arrow and data not shown). Therefore, our results suggest that Ptpro activity may be involved in regulating Fgf signaling during cerebellar development.

### Ptpro modulates the Fgf signaling pathway

**Fig. 5:**
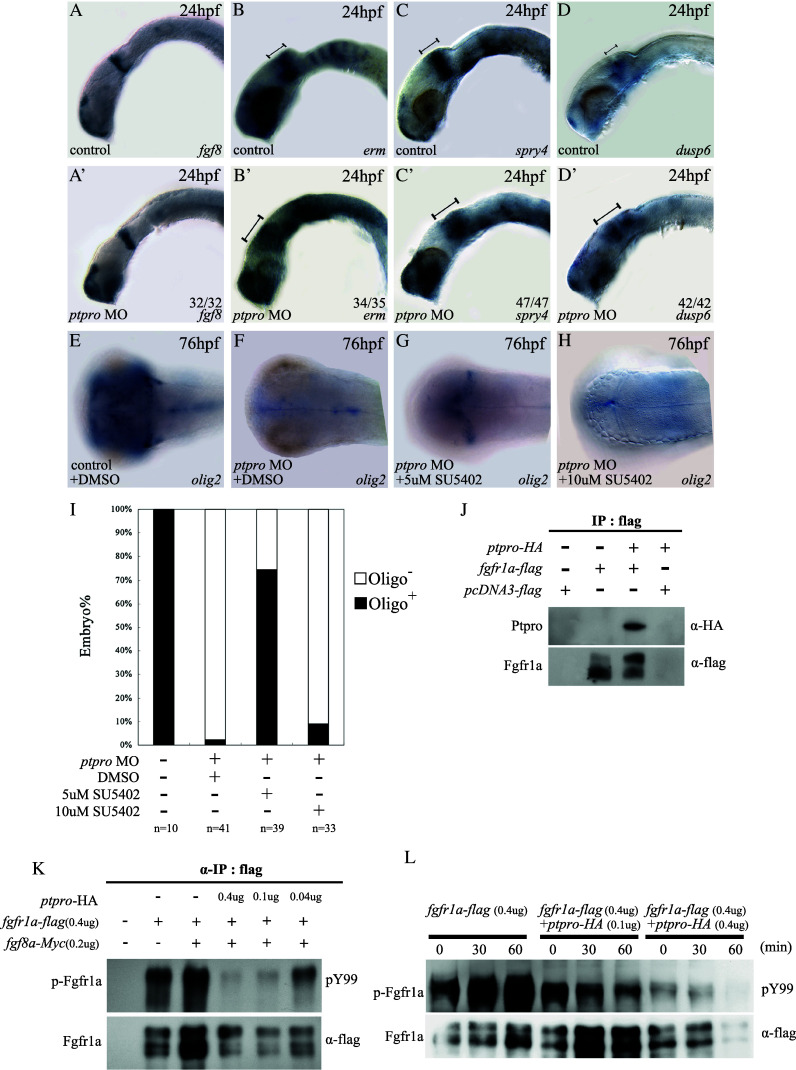
Ptpro modulates the Fgf signaling pathway. **a**–**d**′ Images of WMISH results from control (**a–d**) and *ptpro* MO-injected (**a**′–**d**′) embryos at 24 hpf. **e**–**h** Images of WMISH results from control (**e**) and *ptpro* MO-injected (**f**–**h**) embryos treated with DMSO (**f**) or 5–10 μM SU5402 (refer to “Results“) at 76 hpf. Each specific mRNA detected by WMISH is shown in the *bottom right corner* of each image. Lateral views with the anterior to the *left* and dorsal to the *top* in (**a**–**d**) and (**a**′–**d**′); dorsal views with the anterior to the *left* and *right* to the *top* in (**e** and **h**). **i** Chart showing the fraction of oligo2 positive embryos injected with different combinations of the *ptpro* MO and SU5402. **j** Image showing co-immunoprecipitation (IP) analysis of the interaction between zebrafish Ptpro and Fgfr1a. An anti-flag IP was conducted with cells transfected with either an empty vector, or the flag-tagged *fgfr1*a plasmid, or the HA-tagged *ptpro* plus flag-tagged *fgfr1a* plasmids as indicated at the top of the image. **K** Top image showing the tyrosine phosphorylation analysis of ectopically expressed Fgfr1a detected with anti-phosphotyrosine antibodies (‘pY99’). Anti-flag IP was conducted with cells transfected with either empty vectors, or 0.4 μg flag-tagged *fgfr1a* plasmid combined with 0–0.4 μg HA-tagged *ptpro* and 0–0.2 Myc-tagged *fgf8a* plasmids as indicated at the *top* of the image. The *bottom image* shows the amount of total Fgfr1a precipitated with anti-flag antibodies in each reaction. **l** *Top image* shows the tyrosine phosphorylation analysis of ectopically expressed Fgfr1a under fgf8 stimulation. Anti-flag IP was conducted with cells transfected with 0.4 μg of the Flag-tagged *fgfr1a* plasmid combined with 0–0.4 μg of HA-tagged *ptpro*, followed by 0, 30, or 60 min of fgf8 stimulation as indicated at the *top* of the image. *Bottom image* shows the amount of total *fgfr1a* precipitated with anti-flag antibodies in each reaction

A previous report showed that Fgf signaling mediates forebrain and cerebellar formation [44]. For example, *fgf3*, *fgf8*, and *fgf19* are required for forebrain formation and patterning of the telencephalon [45–48]. In addition, *fgf8* was shown to play roles in IsO activity [49, 50], cell survival [50], and cerebellar development [18, 21, 50, 51] in vertebrates. Zebrafish *ace* (*acerebella*) mutants lack IsO and cerebellar formation during embryonic development due to the loss of Fgf8 activity [18, 51]. Because our data shown above suggest that Ptpro might regulate *ephrinA* expression through modulating Fgf signaling in *ptpro* morphants (Fig. 4f′), we speculated that Ptpro might function in dephosphorylating Fgf receptors (Fgfrs) during embryonic brain development. Previous reports indicated that *erm, spry4, dusp6/mkp3*, and *sef* are all under the control of the Fgf signaling pathway. Up- or downregulating Fgf signaling will respectively increase or reduce expressions of these genes [51–55]. To investigate whether Ptpro is involved in modulating the Fgf signaling pathway, we examined expressions of *erm*, *spry4, dusp6*, and *sef* in *ptpro* MO-injected embryos at 24 hpf. Results showed that while *fgf8* expression was not notably altered within the IsO region in *ptpro* morphants (Fig. 5a, a′), expression domains of *erm* and *spry4* were slightly expanded to the metencephalon region (Fig. 5b, b′, c, c′). In addition, *dusp6* expression significantly increased in the IsO and expanded to the metencephalon in *ptpro* morphants (Fig. 5d, d′). Hence, altered expressions of these Fgf signaling response genes in *ptpro* morphants provided evidence supporting our hypothesis that Ptpro may regulate the Fgf signaling pathway by dephosphorylating the Fgfrs.

### Cerebellum formation was partially rescued by inhibiting Fgf signaling in *ptpro* morphants

Previous studies indicated that loss of *fgf8* signaling activity in *ace* mutants and low-level overexpression of *spry4*, a feedback-induced antagonist of Fgf signaling, can result in loss of cerebellar formation in zebrafish [18, 51]. *The dusp6*, an inducible antagonist of Fgf signaling, acts in a negative feedback loop to attenuate Fgf signaling in mice [56]. Results of our analyses indicated that *dusp6* expression was increased, and both *spry4* and *dusp6* expressions were partially expanded to the metencephalon by downregulating Ptpro by MO injection (Fig. 5c′, d′). This observation suggests that perturbing Ptpro expression might increase Fgf signaling, causing overexpression of Fgf signaling negative feedback genes within the MHB that eventually led to Fgf signaling attenuation in *ptpro* morphants during cerebellar formation. According to this hypothesis, we used the Fgfr-specific inhibitor, SU5402, to attenuate Fgf signaling to examine whether the cerebellar defect could be rescued in *ptpro* morphants. Previously, SU5402 has been used to block Fgf signaling in zebrafish embryos and was shown to markedly reduce Fgf signaling target gene expression in wild type or transgenic zebrafish at doses ranging from 5–10 μM [52, 53, 57]. By examining the cerebellum-specific marker *olig2* expression, we observed no change in control embryos (5E, phenol red injection plus DMSO treatment); partial rescue of cerebellar cell specification in *ptpro* morphants treated with 5 μM SU5402 (Fig. 5g), but not in DMSO-treated and 10 μM SU5402-treated (complete block) *ptpro* morphants (Fig. 5f, h, i) Therefore, these results support our hypothesis that Ptpro contributes to cerebellar formation by regulating Fgf signaling.

Previously, it was shown that *fgfr1* is expressed within the IsO region and is required for establishing the MHB in mice [58, 59]. In addition, Fgfr1a is required for Fgf8 signaling activation at the MHB and during cerebellar formation in zebrafish [21, 51, 60]. Therefore, we hypothesized that Fgfr1a might be regulated by Ptpro in Fgf signaling-mediated cerebellar development. We examined the molecular interaction between Ptpro and Fgfr1a that were ectopically co-expressed in cultured cells by co-immunoprecipitation (Co-IP). Flag-tagged Fgfr1a and HA-tagged Ptpro were expressed in 293T cells, and immunoprecipitation was performed with or without the anti-Flag antibody. Results showed that HA-tagged Ptpro was co-precipitated with Flag-tagged Fgfr1a (Fig. 5j) but no HA-tagged Ptpro was precipitated when *ptpro*-HA was expressed , indicating that Ptpro specifically interacts with Fgfr1a within cultured cells.

In order to further verify that Ptpro is able to dephosphorylate Fgfr1a in 293T cells, we employed a Co-IP experiment to precipitate Flag-tagged Fgfr1a and then examined tyrosyl phosphorylation of Fgfr1a by an anti-Tyr (PY99) antibody. Our results showed that when Flag-tagged Fgfr1a was co-expressed with HA-tagged Ptpro, the tyrosyl phosphorylated level of Fgfr1a was markedly reduced in a dose-dependent manner (Fig. 5k, lanes 3–5). In addition, we examined the effects of the co-expression of Ptpro on tyrosyl phosphorylation of Fgfr1a when stimulated with Fgf8a-conditioned medium in living cells. We first confirmed that extracellularly applied Fgf8a-conditioned medium markedly enhanced the tyrosyl phosphorylation of fgfr1a in a time-dependent manner (Fig. 5l, lanes 1–3). When Ptpro was co-expressed, enhancement by Fgf8a-conditioned medium was partially or markedly suppressed, depending on the expression level of Ptpro (Fig. 5k, lanes 4–6 and 7–9). Therefore, these results strongly suggest that Fgfr1a is a physiological substrate for Ptpro, and Ptpro can regulate Fgf signaling by dephosphorylating Fgfr1a.

## Discussion

In this report, we demonstrated that the full-length zebrafish Ptpro is highly conserved compared to mouse and human Ptpros, suggesting that a conserved functional mechanism might exist among different vertebrates. In addition, zebrafish *ptpro* transcripts were primarily expressed in the embryonic and adult CNS. With similar expression appearances of *ptpro* transcripts in adult mouse and chick brains, it is conceivable that Ptpro may be required to maintain proper functions of the adult vertebrate brain [16, 61]. A functional study of Ptpro in the adult vertebrate brain is required to validate this hypothesis.

Utilizing an antisense MO to inhibit translation or splicing of the endogenous *ptpro* transcripts, we found that a lack of Ptpro activity during early embryonic development caused patterning defects in the developing brain. A previous analysis of *ptpro* knockout mice reported that loss of endogenous Ptpro activity caused defects in neuronal process outgrowth and a reduction in peptidergic nociceptive neurons in DRGs in the spinal cord [15]. In our analyses, we found that transcriptional expressions of the patterning gene, *dlx2*, the motor neuron marker, *isl1,* and the GABAergic interneuron marker, *gad1,* were all reduced in the telencephalon of *ptpro* morphants (Fig. 3b, d, e). On the other hand, expressions of the patterning gene, *emx1*, in the dorsal pallial domain and of the post-mitotic neuronal marker, *huC*, both increased in the telencephalon of *ptpro* morphants (Fig. 2A, G). Similar patterning and cell specification defects were observed in the diencephalon and midbrain of *ptpro* morphants when expressions of *isl* in the hypothalamus and *shh* and *oligo2* in the dorsal thalamus were reduced, while the expression of *huC* greatly increased in these regions (Fig. 3c, d, f–h). Therefore, our experimental results indicate that Ptpro activity is required for proper neuronal cell fate determination in the developing zebrafish embryonic brain. However, whether the increase in *huC*-positive neurons in *ptpro* morphants was caused by abnormal gene expression control or by abnormal early onset of neuronal differentiation cannot be distinguished by the analyses in this report. Further analyses of the differentiation processes of these neurons will provide clues for answering this question. We also noted that there was abnormal development of *huC*-positive neurons in the cerebellum of *ptpro* morphants (Fig. 4h′). Subsequent analyses indicated that despite the upper rhombic lip marked by *atoh1.1* transcripts being successfully specified in morphants at 52 hpf (Fig. 4a′), specification of the cerebellum was abnormal at later developmental stages as demonstrated by reduced expressions of the patterning genes, *evx1* and *pax2a*, at 72 hpf (Fig. 4b, c), and reduced expressions of the granule cell marker, *neuroD*, and Purkinje cell marker, *pvalb7*, at 96 hpf (Fig. 4d, e). These results suggest that loss of Ptpro activity did not perturb early development of the MHB region, but in later stages, development of the cerebellum that is derived from the MHB and surrounding tissues requires the presence of Ptpro activity.

Fgf signaling activity was previously demonstrated to play a decisive role during zebrafish embryonic cerebellar development through activating expression of the downstream *ephrin A* gradient [20, 21]. We found that in *ptpro* morphants, *efnA5* expression in the posterior midbrain and MHB region was greatly reduced but was not completely below a detectable level as what was previously observed in *fgf8* (ace) mutants [20], indicating that Fgf signaling was partially activated in *ptpro* morphants in early developmental stages. Increased expression of the Fgf signaling response gene, *dusp6*, and defective development of the cerebellum in *ptpro* morphants suggest that abnormal cerebellar development in *ptpro* morphants might have resulted from perturbed Fgf signaling activity. This hypothesis was subsequently confirmed by experimental results showing that specification of *oligo2*-positive neurons was partially rescued in *ptpro*-deficient embryos treated with an Fgf signaling antagonist (Fig. 5e–h). In addition, our affinity pull-down assays and tyrosyl phosphorylation level evaluation results indicated that Ptpro physically interacts with Fgfr1a (Fig. 5j), and dephosphorylates Fgfr1a in vitro in a dose-dependant manner (Fig. 5k–l). Therefore, these experimental results strongly suggest that defective Fgfr1 dephosphorylation might account for the abnormal cerebellar specification in *ptpro*-deficient embryos. In addition, the proper specification of upper rhombic lips and the presence of *spry4* and *dusp6* transcripts in *ptpro* morphants provide strong evidence supporting the hypothesis that Fgf signaling activity was initially present in *ptpro* morphants in order to establish the molecular identity of the cerebellum primordium. However, at later developmental stages, the loss of Ptpro activity likely perturbed the Fgf signaling activity in *ptpro* morphants that eventually led to a defective cerebellar development. The incomplete rescue of cerebellar defects after introducing Fgf signaling antagonists into *ptpro* morphants suggests the possibility that the perturbed activity of the Fgf signaling machinery in *ptpro*-deficient embryos gradually evolved to an irreversible defective mode, where after only a particularly short transition period, the introduction of Fgf signaling antagonists could alleviate part of the cerebellar specification defects caused by loss of Ptpro activity. Both Ptpro and SU5402 exert their influences on Fgf signaling through Fgfrs. However, unlike the SU5402 that merely negatively regulates Fgfr activity, loss of Ptpro activity at the initial stage would increase both the activity of Fgfr signaling and the negative feedback signaling (the increase of *dusp6*) at the same time. Therefore, why MO phenotype can be rescued slightly by SU5402 in a short developmental window is because the addition of SU5402 slows down the abnormal elevation of the Fgf negative feedback factors in the *ptpro* morphants.

Previous studies of Fgfrs indicated that ligand binding triggers the self-phosphorylation of Fgfr cytoplasmic domains which then promotes the dimerization of Fgfr monomers and leads to the activation of downstream signal transduction inside receiving cells [23, 24]. Therefore, to explain the results reported in this article, we propose that modulation of Fgfr monomer turnover on plasma membranes by Ptpro is required to maintain the proper level of Fgf signaling activity during embryonic cerebellar development. The dephosphorylation of ligand-bound Fgfr dimers by Ptpro releases Fgfr monomers and promotes the reactivation of Fgfr monomers to be competent again for ligand binding. When Ptpro activity was absent, the release of Fgfr monomers from ligand-bound dimers was inhibited, and constitutively activated receptors induced conformational inactivation or pre-mature degradation of Fgfrs that eventually led to depletion of ligand-free Fgfr monomers on plasma membranes. This model of Ptpro function can explain the initial presence of Fgf signaling activity in *ptpro*-deficient embryos, and why at a later developmental stage, loss of Ptpro activity caused loss of the ephrinA gradient, which was similar to a previously observed phenotype in *fgf*-deficient acerebellar embryos [21]. In addition, this model could explain why the lack of Ptpro activity slightly elevated the initial Fgf signaling activity and caused some of the cerebellar developmental defects similar to a previously observed phenotype in low-level *spry4*-overexpressing embryos [51], because failure to dephosphorylate Fgfr dimers would allow constitutively activated receptors to remain on the membrane before they are subjected to inactivation or degradation. Furthermore, inhibition of Fgf signaling activity resulted in the incomplete rescue of cerebellar defects in *ptpro* morphants because later cerebellar developmental defects were caused by the abnormal depletion of Fgfr1a on cell membranes.

Taken together, our experimental results demonstrated that Ptpro activity is required for controlling zebrafish embryonic brain development. Specifically, our analyses suggest that dephosphorylating Fgfr1a dimers by Ptpro is crucial for the cerebellum development in the zebrafish embryonic brain, presumably for helping to maintain the proper levels of Fgfr monomers on plasma membranes. Nevertheless, our analyses do not provide enough evidence to rule out the possibility that other molecules and mechanisms may contribute to cerebellar and other developmental defects in *ptpro* morphants. Previously, two groups reported that Wnt signaling plays roles in vertebrate cerebellar development, and a study by Kim et al. showed that Ptpro might serve as a Wnt receptor for signal transduction [8, 39, 62]. In addition, more than just *fgfr1* is expressed in the MHB and surrounding tissues. Therefore, study efforts focusing on the relationship between Ptpro and Wnt signaling, or on the interactions between Ptpro and other Fgfrs are inevitably required to generate a more-comprehensive picture of the function of Ptpro during cerebellar development in the vertebrate embryonic brain.

## Materials and methods

### Zebrafish care and embryo collection

The zebrafish wild-type AB line was raised and maintained at 28.5 °C. Embryos were harvested and staged as previously described [63].

### Whole-mount in situ hybridization (WMISH)

Previously described RNA probes that were utilized in this report included *atoh1a, dlx2, dusp6, efnA5a, efnA5b, emx1, erm, evx1, fgf8, gad1, huC, is11, neuroD, olig2, pax2a, pvalb7, shh*, and *spry4* [21, 32, 35, 40, 47, 54, 64]. The zebrafish cDNA clone, zgc:158179, was purchased from IMAGE (Human Genome Center, Lawrence Livermore National Laboratory, Livermore, California, USA) and the full complementary cDNA fragment was subcloned into the pGEMT easy vector (Promega, Madison, WI, USA) for subsequent application. Specific primers for *ptpro*: *ptpro*-3′UTR (see above) and another probe (*ptpro*-EC-F: GCA CTG GTT GTC AGG TGT GTG TTA C-3′ and *ptpro*-EC-R: GTG GGC ATC ACA GCA GGC ATC ACA G-3′) were used to recognize sequences coding the *ptpro* extracellular region. For probe synthesis, plasmids were linearized and transcribed with T7 or SP6 RNA polymerase (Promega) as previously indicated in each corresponding reference (see above). The WMISH was performed as previously described [65]. For probe detection, NBT/BCIP (blue) or Fastred (red) (Roche, Mannheim, Germany) was used as the substrate for alkaline phosphatase. Images of NBT/BCIP-stained samples were acquired using an AxioPlan-2 microscope (Carl Zeiss Microscopy, Jena, Germany) and SPOT-RT color (Diagnostic Instruments, Sterling Heights, USA).

### Reverse-transcription polymerase chain reaction (RT-PCR)

Total RNA was isolated from different developmental stages and various tissues of adult zebrafish using the TRIzol*®* reagent (Invitrogen, Carlsbad, CA, USA). An RT-PCR was performed as previously described [66]. The PCR amplifications were performed with the following zebrafish *ptpro* primers (*ptpro*-3′UTR-F, 5′- GAG TTG TCA TCA GTG TTG AAC ACA CAC -3′ and *ptpro*-3′UTR-R, 5′- AGA AAC ATT CAC AGC GGT GCA GAT ACA-3′). Zebrafish *β*-*actin* primers (*zACT*-F, 5′-GTG CTA GAC TCT GGT GAT GGT GTG-3′ and *zACT*-R, 5′-GGT GAT GAC CTG ACC GTC AGG AAG-3′) and *GAPDH* primers *(zGAPDH*-F, 5′-TGG GTG TCA ACC ATG AGA AA-3′ and *zGAPDH*-R, 5′-AAC CTG GTG CTC CGT GTA TC-3′) were used for the internal control. Primers F1 (5′-GAG CGC AGT TCC ATC ACT CGC TAT TG-3′), R1 (5′-GTG TTC TGC CGT CGG TCA TCA GGC-3′), F2 (5′-GTG TTG CTG TCC TCC GTC CGG CTG-3′) and R2 (5′-CGT TCA TCT GTG TGA CCC AGT TTC GC-3′) were used for examining the effectiveness of the *ptpro* splicing MO.

### Morpholino oligonucleotide (MO) injections

The MOs for inhibiting zebrafish *ptpro* translation (MO = MO^ATG^, sequence listed in Fig. 3a) and for inhibiting *ptpro* splicing (MO^SB^, 5′-TGA ACG GAA TAT GCA CGC ACC TGA A-3′) were designed following the rules recommended by Gene Tools (Philomath, OR, USA). Embryos injected with 1 μl of 1 % phenol red (Sigma, St. Louis, MO, USA) were collected for use as control. For MO effectiveness tests, the 25-bp MO target sequence was cloned upstream of the enhanced green fluorescent protein (EGFP) open reading frame (ORF) in the pcDNA3.1-EGFP reporter plasmid (Invitrogen) to create the *zptpro*-*egfp* construct. As a control, an MO target sequence bearing five mismatches was introduced into the pcDNA3.1-EGFP reporter plasmid to create the MM-*zptpro*-*egfp* construct. One- to two-cell embryos were microinjected with 0.25–4 ng of morpholino alone, or co-injected with 2 ng of the reporter plasmids. The *p53* MO at 0–0.1 ng was used to examine the off-targeting effect of the *ptpro* MO [26]. At least three independent replicates were run for all experiments.

### Immunoprecipitation

The 293T cells used in this study were obtained from the American Type Culture Collection (Manassas, VA, USA). The 293T cells were maintained at 37 °C in minimal essential medium-alpha medium, 10 % fetal bovine serum, 100 mg/ml streptomycin, and 100 U/ml penicillin. Plasmid transfection and cell collection were done with standard procedures as described previously [67]. Transfected 293T cells collected from one 6-well plate were lysed in 0.1 ml immunoprecipitation lysis buffer (150 mM NaCl, 20 mM HEPES (pH 7.2), 10 mM NaF, 1 mM EDTA, 0.5 % NP-40, 1 mM Na3VO4, 1 mM PMSF, and 1 mM DTT), then sonicated for 10 s three times using a UP50H machine at an 80 % power level (Dr. Hielscher, Teltow, Germany). The lysate was centrifuged at 14,000 *g* for 15 min at 4 °C. Total protein at 0.4 mg from the supernatant was pre-absorbed with protein A/G Sepharose beads (Santa Cruz Technology, Santa Cruz, CA, USA). The pre-absorbed solution was centrifuged at 2,500*g* for 1 min at 4 °C, then the supernatant was incubated with 1 μg of an anti-flag M2 antibody (Sigma) at 4 °C overnight. The protein-antibody complex was immunoprecipitated with the addition of protein A/G Sepharose beads and incubated for 2 h. Beads were washed five times with phosphate-buffered saline followed by boiling in gel loading sample buffer for 5 min to denature and release the bound proteins. After sodium dodecylsulfate polyacrylamide gel electrophoresis (SDS-PAGE), the denatured proteins were analyzed by Western blotting with the following antibodies: a 1/10^4^ dilution of anti-flag M2 (Sigma), a 1/10^4^ dilution of anti-HA (Santa Cruz), a 1/10^4^ dilution of PY99 (Santa Cruz), a 1/5,000 dilution of anti-ptpro (Abcam, UK), a 1/10^4^ dilution of anti-rabbit IgG (H + L), horseradish peroxidase (HRP) conjugated, and 1/10^4^ anti-mouse IgG (H + L), and HRP conjugated (Jackson ImmunoResearch Laboratories, West Baltimore Pike West Grove, PA, USA). To prepare conditioned medium, 293T cells (2.2 × 10^6^) were plated on 10 cm dishes (Nunc, Roskilde, Denmark), then transfected with the pcDNA3-fgf8a-myc plasmid at 1 day and grown in serum-free Dulbecco’s modified Eagle medium for 2 days. The conditioned medium was collected, filtered though a 0.45 μm syringe filter (Pall, Port Washington, USA), and immediately used for Fgf-stimulated experiments.

### Fgf signaling assay by SU5402

Collected embryos were grown in embryo medium [63]. At 22 h post-fertilization (hpf), embryos subjected to Fgfr inhibition were incubated in 5–10 μM SU5402 (Calbiochem, San Diego, CA, USA) until embryos were fixed. Control embryos were incubated with equal amounts of solvent (0.5 % DMSO). Embryos were collected and fixed at 76 hpf followed by the WMISH analysis using the cerebellum-specific probe, *olig2* [32]. Successful rescue of cerebellum defects in *ptpro* morphants by the SU5402 were scored qualitatively by the emerging expression of *olig2* in the developing cerebellum that normally was missing from the *ptpro* morphants.

### Electronic supplementary material

Below is the link to the electronic supplementary material.

Supplementary material 1 **Sequence alignment between zebrafish, mouse, and human Ptpros.** The amino acid sequence of zebrafish Ptpro was aligned with mouse and human PTPROs using ClustalW2 (http://www.ebi.ac.uk/Tools/msa/clustalw2/). Residues marked with shading are conserved among the PTPRO/Ptpro proteins, and dashes indicate the gaps introduced to optimize the alignment. The signal peptide, putative fibronectin type III-like domain, transmembrane domain, tyrosine phosphatase domain, and YxNΦ motif in the C-terminal region are highlighted with brackets at the top of each domain. The accession numbers are NP_001077283 for zebrafish, NP_035346 for mouse, and NP_109592,1 for human Ptpro/PTPROs. Percentages of identity and similarity between zebrafish Ptpro with mouse and human PTPRO are listed at the ends of the sequences. (PDF 1873 kb)

Supplementary material 2 **The** ***ptpro*** **splicing MO also induced specific brain defects.** (A) Schematic drawing showing the positions for the splicing MO and the primers used for detecting the *ptpro* derived RNA species by RT-PCR. (B) Representative images showing various degrees of abnormalities in the *ptpro* translation blocker MO (MO^atg^) or splice blocker MO (MO^SB^)-injected embryos at 24 hpf. Anterior side is on the left and dorsal side is on the top. (C) Chart showing the distributions of each phenotypic category of embryos injected with various doses of the *ptpro* MO^atg^ or MO^SB^. (D) Images from the RT-PCR analyses of the control (C) of MO^SB^ injected embryos. Images of WMISH results from ptpro MO^atg^- (E-F) and *ptpro* MO^SB^-injected (E’-F’) embryos at various stages as indicated in the top right corner of each image. Each specific mRNA detected by WMISH is shown in the bottom right corner of each image. Dorsal views with the anterior to the left and right to the top in (E-F) and E’-F’) (PDF 602 kb)
