# Supplementary figures and images for: Protein tyrosine phosphatase receptor type O (Ptpro) regulates cerebellar formation during zebrafish development through modulating Fgf signaling
Source: Cell Mol Life Sci. 2013 Jan 30;70(13):2367–81. doi: 10.1007/s00018-013-1259-7 (PMC3676743; doi:10.1007/s00018-013-1259-7)

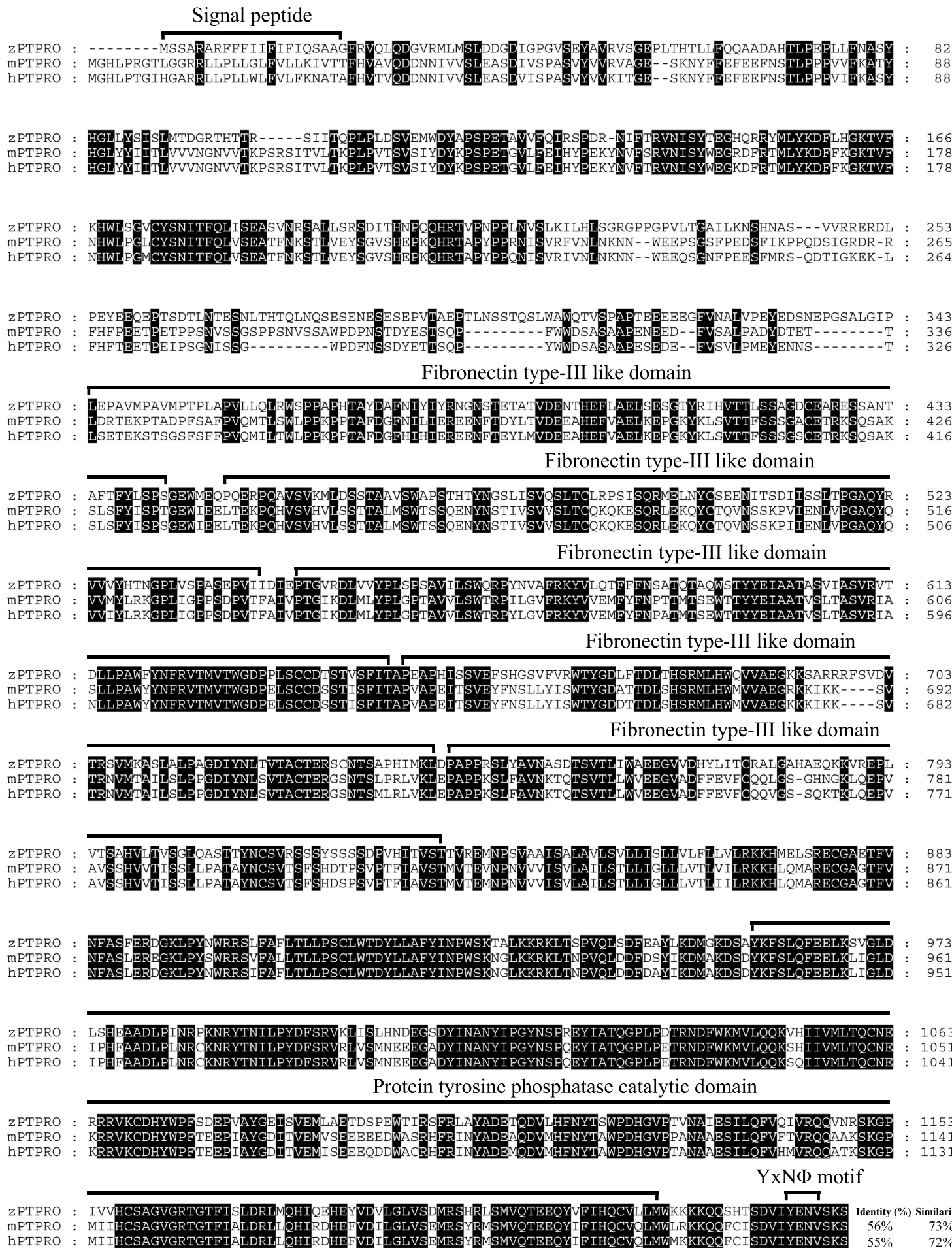

Supplemental Figure 1.

Supplement: Supplementary file 1 — Supplementary material 1 Sequence alignment between zebrafish, mouse, and human Ptpros. The amino acid sequence of zebrafish Ptpro was aligned with mouse and human PTPROs using ClustalW2 (http://www.ebi.ac.uk/Tools/msa/clustalw2/). Residues marked with shading are conserved among the PTPRO/Ptpro proteins, and dashes indicate the gaps introduced to optimize the alignment. The signal peptide, putative fibronectin type III-like domain, transmembrane domain, tyrosine phosphatase domain, and YxNΦ motif in the C-terminal region are highlighted with brackets at the top of each domain. The accession numbers are NP_001077283 for zebrafish, NP_035346 for mouse, and NP_109592,1 for human Ptpro/PTPROs. Percentages of identity and similarity between zebrafish Ptpro with mouse and human PTPRO are listed at the ends of the sequences. (PDF 1873 kb) [file 18_2013_1259_MOESM1_ESM.pdf]

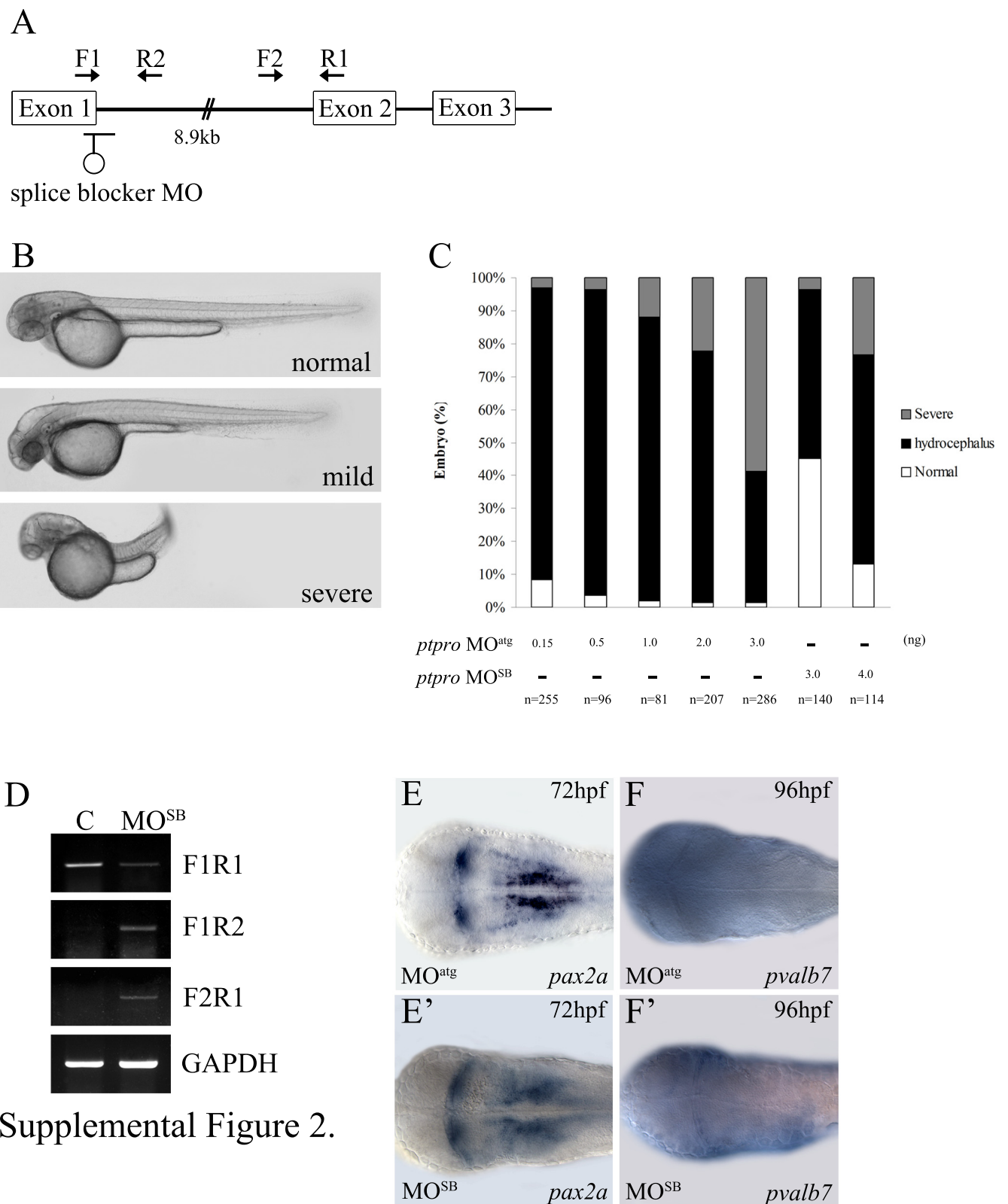

Supplement: Supplementary file 2 — Supplementary material 2 The ptpro splicing MO also induced specific brain defects. (A) Schematic drawing showing the positions for the splicing MO and the primers used for detecting the ptpro derived RNA species by RT-PCR. (B) Representative images showing various degrees of abnormalities in the ptpro translation blocker MO (MOatg) or splice blocker MO (MOSB)-injected embryos at 24 hpf. Anterior side is on the left and dorsal side is on the top. (C) Chart showing the distributions of each phenotypic category of embryos injected with various doses of the ptpro MOatg or MOSB. (D) Images from the RT-PCR analyses of the control (C) of MOSB injected embryos. Images of WMISH results from ptpro MOatg- (E-F) and ptpro MOSB-injected (E’-F’) embryos at various stages as indicated in the top right corner of each image. Each specific mRNA detected by WMISH is shown in the bottom right corner of each image. Dorsal views with the anterior to the left and right to the top in (E-F) and E’-F’) (PDF 602 kb) [file 18_2013_1259_MOESM2_ESM.pdf]
